# Changes to glaucoma surgery patterns during the coronavirus disease 2019 pandemic: a shift towards less invasive procedures

**DOI:** 10.1080/07853890.2022.2157474

**Published:** 2022-12-28

**Authors:** Natalia Dub, Kinga Gołaszewska, Emil Saeed, Diana Anna Dmuchowska, Iwona Obuchowska, Joanna Konopińska

**Affiliations:** Department of Ophthalmology, Medical University of Bialystok, Bialystok, Poland

**Keywords:** Glaucoma surgery, SARS-CoV-2 pandemic, minimally invasive glaucoma surgery, glaucoma surgery patterns, COVID-19, trabeculectomy, canaloplasty, iStent

## Abstract

**Purpose:**

The aim of the study was to compare the quantity, type of glaucoma surgeries, and the disease stage before and during the coronavirus disease 2019 (COVID-19) pandemic.

**Methods:**

This was a retrospective, single-centre consecutive case series that included medical records of patients who underwent glaucoma surgery at the University Hospital in Białystok between 4 September, 2018, and 3 March, 2020 (pre-pandemic group) and compared it with patients treated between 4 March, 2020, and 4 September, 2021 (pandemic group). Adult patients with primary or secondary open-angle or closed-angle glaucoma who underwent surgery were included in this study. Finally, 534 operated eyes (362 and 172 eyes operated on before and during the pandemic, respectively) were examined.

**Results:**

The number of glaucoma surgeries dropped by 50% during the pandemic compared to a similar pre-pandemic period, with a significant difference in the kind of procedure between the two groups (*p* < 0.001). The most common procedures in the pre-pandemic group were Ex-Press implantation (33.7%) and trabeculectomy (31.5%). Within the pandemic group, half of the eyes underwent trabeculectomy (50.0%), followed by Preserflo microshunt (11.6%), iStent (8.7%), and transscleral cyclophotocoagulation (TSCP) (8.7%). A significant difference in the average intraocular pressure was revealed among patients who qualified for surgery.

**Conclusion:**

The COVID-19 pandemic is associated with a decrease in the number of extended antiglaucoma procedures and an increase in the number of short procedures performed, such as TSCP and minimally invasive glaucoma surgery.

**Key Messages**
- Our study has shown the negative impact of the COVID-19 pandemic in reducing the number of antiglaucoma procedures.
- The number of glaucoma surgeries dropped by 50% during the pandemic compared to those in a similar pre-pandemic period, and the type of performed procedures has changed.
- The COVID-19 pandemic is associated with a decrease in the number of combined antiglaucoma procedures, in opposite: the number of minimally invasive glaucoma surgeries increased due to safety reasons.

## Introduction

Glaucoma is the leading cause of blindness worldwide, affecting 76 million people in 2020 [1]. This number is expected to increase by 40 million in the next few years. In Poland, with a population of almost 38 million, the number of patients affected by glaucoma has been estimated at 800,000 [2]. Glaucoma treatment remains a challenge, and treatment discontinuation may result in an increased number of patients with irreversible vision loss.

The first unexplained case of acute pneumonia occurred in November 2019 in Wuhan, China [3] and was soon thereafter associated with severe acute respiratory syndrome coronavirus 2 (SARS-CoV-2); the resulting disease is now known as the coronavirus disease 2019 (COVID-19) [4]. Due to the rapid and global spread of the disease, the World Health Organization proclaimed the outbreak as a pandemic on 11 March, 2020. In Poland, the first case of COVID-19 was reported on 4 March 2020, and the first national lockdown was declared on 23 March 2020 [5,6].

The pandemic has forced a reorganization of healthcare systems worldwide, including the mobilization and prioritization of resources to treat COVID-19 patients. To limit disease spread and to protect resources, many ophthalmic societies provided guidelines for classifying procedures as urgent and non-urgent [7], with the latter group’s procedures being postponed [8]. Certain ophthalmology departments were converted into COVID-19 wards, admitting only SARS-CoV-2-positive patients, and limiting access to ophthalmic treatment. The fear of infection among older adults and patients with comorbidities resulted in delays when seeking treatment for cataracts or chronic glaucoma [9, 10].

The Ophthalmology Department of Medical University in Białystok, Poland, is a reference centre for most patients affected by glaucoma in Podlasie voivodeship (population of approximately 1.2 million people). It is the only hospital in this region that offers surgical treatment for glaucoma. Despite the restrictions placed on elective surgeries during the COVID-19 pandemic, glaucoma surgeries were classified as urgent and were regularly performed. The Royal College of Ophthalmology provided recommendations for qualifying glaucoma patients for surgical treatment [11], including those needing immediate and non-immediate surgery, based on the intraocular pressure (IOP), extent of vision and visual field (VF) losses, disease progression rate, and suitability of other treatments. However, these recommendations did not address surgery or anaesthesia types, both of which remained at the surgeon’s discretion.

In addition to some generalized recommendations on safety during the pandemic [12], there has been a few studies on the impact of the COVID-19 pandemic on ophthalmology departments and ophthalmic outpatient clinical activities [10, 13–20]. Rajendrababu et al. [21] made a comparison between emergency glaucoma patients treated in a tertiary eye care centre in Madurai, South India, both before and during the COVID-19 lockdown. Moreover, Holland et al. [22] examined the impact of pandemic-related changes in glaucoma surgical practices in the United Kingdom. A separate study from the Tongji Hospital in Wuhan City, China, reported that glaucoma surgery was the most frequent subspecialty surgery performed during the COVID-19 pandemic [23].

Given the lack of evidence on the impact of the COVID-19 pandemic on glaucoma treatment, this study aimed to compare surgical glaucoma care before and during the pandemic, including the quantity and type of glaucoma procedures performed and the stage of the disease. To the best of our knowledge, our paper is the first report from a Central Eastern European referral centre and might help to plan glaucoma surgery strategies in other cities and countries in this geographical region.

## Materials and methods

This was a retrospective, single-centre consecutive case series audit that included patients with the International Classification of Diseases and Tenth Revision final diagnosis codes of B11 and B98, which encompasses glaucoma surgery. Data of patients who underwent glaucoma surgery at the University Hospital in Białystok between 4 September 2018 and 3 March 2020 were extracted from their medical records and compared with those of glaucoma patients treated between 4 March 2020 and 4 September 2021. All adult patients with primary or secondary open-angle or closed-angle glaucoma who underwent glaucoma surgery in the periods of interest were included in this study. Finally, 534 operated eyes (362 and 172 eyes operated on before and during the pandemic, respectively) were examined. All patients from both groups underwent a full ophthalmic examination prior to surgery to confirm the diagnosis of glaucoma. The ophthalmic examination included the assessment of best corrected visual acuity (BCVA) using the Snellen charts; IOP measurement by Goldmann applanation tonometry; assessment of the anterior segment of the eye by slit lamp, including ocular lens opacification severity, as determined by the Lens Opacification Classification Scale III; gonioscopy by Goldmann three-mirror lens; assessment of optic nerve disc by Volk lens, considering glaucoma-specific changes; and optical coherence tomography (OCT) (Heidelberg Engineering Heidelberg, Germany) of the optic nerve head and retinal nerve fibre layer (RNFL) [24]. Indications for surgery were defined as follows: (1) age ≥ 18 years, (2) progression of glaucoma in VF examination/OCT, and (3) failure to achieve target IOP with maximally tolerated topical IOP-lowering treatment or intolerance to drugs. The main criteria for glaucoma progression was the deterioration in mean deviation or RNFL thickness seen in two or three subsequent VF/OCT examinations.

In the period before the COVID-19 pandemic, each patient routinely underwent the 24-2 visual field examination (Humphrey Field Analyzer, Zeiss Medical Technology,  Dublin, CA). During the COVID-19 pandemic, when social distancing measures were introduced, examinations were conducted according to the disease spread containing guidelines [25]. Consequently, visual field examination was performed only in cases in which the OCT measurements of the optic nerve head and the retinal nerve fibre layer were insufficient to assess the severity of glaucomatous optic neuropathy. The reasons for insufficient OCT examination were: insufficient translucency of the optical centres, problems with patient′s cooperation or lack of proper visual fixation, patients with abnormal optic disc structure due to other than glaucomatous damage (e.g. tilted optic disc), patients with high myopia, and very advanced glaucoma. In cases when OCT was insufficient we used “MD – mean deviation” as the value of the VF to determine the need for surgery. Additionally, in the early stages of glaucoma OCT is more useful in contrast to more advanced cases where VF examination is more reliable [24].

The primary outcomes included the number of glaucoma surgeries performed and pre-surgical IOP values. The secondary outcomes included BCVA values, the extent of glaucomatous damage on OCT findings, and the type of surgery performed.

The study protocol adhered to the principles of the Declaration of Helsinki and was approved by the Bioethics Committee of the Medical University of Białystok (APK.002.87.2021). The informed consent requirement was waived due to the retrospective nature of this study. This study abided by the relevant data protection laws.

### Surgical technique

Surgical procedures were performed as one-day procedures under local retrobulbar anaesthesia by two experienced surgeons (J. K. and E. S.). Patients diagnosed with clinically significant cataracts (according to the Lens Opacification Classification Scale III) underwent a combined procedure of glaucoma surgery with simultaneous phacoemulsification. Patients with open-angle glaucoma qualified for one of the following surgical interventions: trabeculectomy, Ex-Press implantation, canaloplasty, and deep sclerectomy; patients with closed-angle glaucoma qualified for trabeculectomy. Patients with early-stage glaucoma or intermediate glaucomatous neuropathy underwent stent implantation. Additional reason for choosing MIGS was the fact that it requires less postoperative follow-up visits than classical surgeries. In case of MIGS there is no need for additional procedures, i.e.: suturolysis, goniopuncture or needling, which are time-consuming and require many repetitive visits. Traditional penetrating glaucoma surgeries were performed in patients with severe glaucomatous optic neuropathy. In the pandemic group, antimetabolites were switched and mitomycin C 0.4 mg/ml for 2 min was administered during trabeculectomy to maximize the effect of the filtering bleb and reduce postoperative interventions. In prepandemic period, 5-fluorouracil was used intraoperatively (50 mg/ml for 5 min). Other surgical procedures were performed in the same manner in the pre-pandemic and the pandemic group.

During the pandemic, all patients underwent polymerase chain reaction testing for SARS-CoV-2 infection 2 days before the surgery, which was covered by the National Health Fund. Patients were then placed in self-isolation until admission for surgery. Since 1 April 2021, all vaccinated patients and patients that recovered from COVID-19 in the previous 3 months were exempt from the testing requirement.

### Statistical analysis

Statistical analysis was performed using the R package, version 4.1.0 (Foundation for Statistical Computing, Vienna, Austria). Nominal variables are presented as counts (%), while continuous variables are presented as mean ± standard deviation (SD) or median (range), depending on data distribution. The normality of the distribution assumption was assessed using the Shapiro–Wilk test, data skewness, and kurtosis indicators based on the visual assessment of histograms. Between-group comparisons were performed using the Chi-square test and Fisher exact test for categorical variables; the *t*-test and Mann–Whitney *U* test were used for comparisons of continuous variables, depending on data distribution. In addition, the mean or median difference and its 95% confidence interval were calculated. A significance level of *α* = 0.05 was used, and all tests were two-sided.

## Results

A total of 306 and 149 patients were included in the pre-pandemic and pandemic groups, respectively. Sex distribution was similar in both groups (*p* = 0.939). The median age values in the pre-pandemic and pandemic groups were 73.0 and 70.0 years, respectively (*p* = 0.212) (Table 1).

**Table 1.** Clinical characteristics of patients with glaucoma, operated on before and during the COVID-19 pandemic.

	Pre-pandemic group	Pandemic group	*p*-value
Number of patients (operated eyes)	306 (362)	149 (172)	<0.001
Sex, *n* (%)			
Male	130 (42.5)	62 (41.6)	0.939
Female	176 (57.5)	87 (58.4)	
Age, median (range)*, years	73.00 (32–95)	70.00 (32–90)	0.212
Number of surgical procedures	354	172	<0.001
Procedures by type, number (%)			
Trabeculectomy	114 (31.5)	86 (50.0)	<0.001
Canaloplasty	66 (18.2)	9 (5.2)	<0.001
iStent	33 (9.1)	15 (8.7)	
Ex-Press	122 (33.7)	11 (6.4)	
Deep sclerectomy	19 (5.2)	15 (8.7)	
Ahmed valve	0 (0.0)	1 (0.6)	
Microshunt Preserflo	0 (0.0)	20 (11.6)	
TSCP	0 (0.0)	15 (8.7)	
Reoperations	8 (2.2)	0 (0.0)	
Phacoemulsification (overall), number (%)			
Yes	180 (50.8)	69 (40.1)	0.026
No	174 (49.2)	103 (59.9)	

TSCP: transscleral cyclophotocoagulation.

Groups were compared using the chi-square test (sex, phacoemulsification/all procedures/, phacoemulsification/all eyes), Mann–Whitney *U* test (age), and Fisher exact test (procedures by type, trabeculectomy versus other procedures).

The total number of procedures in the pre-pandemic and pandemic groups was 354 (354 eyes in 306 patients) and 172 (172 eyes in 149 patients), respectively. The most common procedure type in the pre-pandemic group was Ex-Press implantation (33.7%) and trabeculectomy (31.5%). Canaloplasty was relatively less common (18.2%). IStent and deep sclerectomy were used in <10% of the pre-pandemic patients. Ahmed valve implantation, Preserflo microshunt, and transscleral cyclophotocoagulation (TSCP) were not performed in patients from the pre-pandemic group. In addition, eight patients in the pre-pandemic group underwent reoperation (including one case of Ex-Press explantation due to extrusion, four cases of trabeculectomy revisions, and three cases of laser goniopuncture after deep sclerectomy).

In the pandemic group, 50.0% of the eyes underwent trabeculectomy, while a Preserflo microshunt was used in 11.6% of the cases. IStent, sclerectomy, and TSCP were each used in 8.7% of the eyes. Ex-Press procedure was performed in 6.4% of the eyes, while canaloplasty was performed in 5.2% of the eyes. One eye underwent the Ahmed procedure. The distribution of procedure types differed between the groups (*p* < 0.001) (Table 1).

Before the pandemic, combined glaucoma surgery with phacoemulsification was performed in 50.8% of all cases; the corresponding value during the pandemic was 40.1% (*p* = 0.026) (Table 1).

Groups were compared using the chi-square test (sex, phacoemulsification/all procedures/, phacoemulsification/all eyes), Mann–Whitney *U* test (age), and Fisher exact test (procedures by type, Trabeculectomy vs other procedures).

The BCVA values were non-normally distributed; thus, they were reported as the median value 0.6 (range 0–1) in both groups (*p* = 0.263). The mean IOP was 22.21 (SD = 7.83) mmHg and 25.16 (SD = 9.48) mmHg in the pre-pandemic and pandemic groups, respectively (*p* < 0.001). There was a between-group difference in the OCT values (*p* = 0.021). The average OCT levels in the pre-pandemic and pandemic groups were 57.77 (SD = 19.63) and 62.51 (SD = 17.41), respectively (Table 2).

**Table 2.** Comparison of best corrected visual acuity, intraocular pressure, and optical coherence tomography findings between patients operated on before and during the pandemic.

	Pre-pandemic group	Pandemic group	MD (95% CI)	*p*-value
BCVA^a^ (Snellen)	0.60 (0–1)	0.60 (0–1)	0.00 (0.00; 0.00)	0.263
IOP (mmHg)	22.21 ± 7.83	25.16 ± 9.48	−2.95 (−4.59; −1.32)	<0.001
OCT	57.77 ± 19.63	62.51 ± 17.41	4.75 (0.72; 8.77)	0.021

^a^Data are presented as mean ± SD or median (range).

MD: mean or median difference (pre-pandemic minus pandemic) with 95% confidence interval (CI). *p*-value – *t*-test or Mann–Whitney *U* test. BCVA: best corrected visual acuity; IOP: intraocular pressure; OCT: optical coherence tomography.

## Discussion

This study has shown the negative impact of the COVID-19 pandemic in reducing the number of surgical procedures. During the pandemic, the number of glaucoma surgeries performed at our clinic was half of those performed before the outbreak despite the fact, that the indication criteria for glaucoma surgery were the same in prepandemic as well as in pandemic group. The reason why the surgeries have dropped by 50% was the less number of patients who arrived to the hospital caused by the fear of infection of COVID-19. This significant drop in number of the surgeries performed was among others due to the fear of patients of having contact with other patients/personnel and due to lower number of referrals from outpatient clinics that were closed or worked in reduced manner along with the phone counselling. Patients who qualified for surgeries during the pandemic had significantly higher IOP values than those who qualified before the pandemic. This may be due to a previously reported decline in adherence to ocular hypotensive medication during the COVID-19 pandemic [26]. However, OCT examinations revealed that an increased IOP did not affect the pathological stage of glaucomatous neuropathy. This may be due to patients not self-administering the IOP-lowering eye drops moreover reduced access to an ophthalmologist; however, the study period was too short to reflect any morphological changes in the optic nerve. We also observed changes in the type of glaucoma surgical procedures conducted during the pandemic. The number of time-consuming procedures including canaloplasty, decreased and that of minimally invasive glaucoma surgeries (MIGS), including Preserflo microshunt implantation and TSCP, increased likely due to the reduced operative time. Moreover, the frequency of combined phacoemulsification and glaucoma surgeries decreased, and that of glaucoma surgery alone increased, likely in an attempt to save time and to avoid a potentially aerosol-generating procedure [25]. This approach is consistent with the guidelines for glaucoma patient care during the COVID-19 pandemic, which recommends single procedures and those that are associated with a relatively short follow-up [20]. The most frequently performed procedure in both periods was trabeculectomy, which is associated with its high success rate [27]. A significant increase in the frequency of MIGS is consistent with the long-term trend in the increasing uptake of minimally invasive procedures, including early-stage glaucomatous neuropathy care. The present findings are consistent with those of Rajendrababu et al. [21] who reported a significant decrease in the number of incisional glaucoma surgeries performed in favour of TSCP (by 2.8 times) and phacoemulsification alone (by 4.3 times). An increase in the number of performed phacoemulsifications as glaucoma procedures may be associated with the increase in lens-induced glaucoma, which has been attributed to as a reduction of elective cataract surgeries. A survey of glaucoma surgeons in the United Kingdom showed a change in patient attitudes toward glaucoma surgery after the pandemic outbreak [22]. Before the pandemic, the most popular procedure, as in our study, was trabeculectomy (87% as the procedure of choice). In the above-mentioned study, up to 60% of the surgeons declared modifications to their glaucoma surgery practice during the COVID-19 pandemic, mainly by reducing the number of performed trabeculectomies and by preferring TSCP as the most common alternative method. The reasons for these changes included a shorter postoperative follow-up period, increased procedural efficiency and safety, improved long-term outcomes, reduced operative time, less anaesthesia burden, and fewer interventions in the postoperative period [22]. Despite these advantages, the long-term hypotensive effects of this method remain unclear.

The reduced number of procedures may also be accounted for by fewer patients electing for treatment due to the fear of infection among older adults and patients with comorbidities. Subathra et al. [15] reported a reduction in patient visits at a tertiary eye care centre in South India during the pandemic; barriers to access included lockdown restrictions, transport problems, and fear of infection. In contrast, Mylona et al. [28], who conducted their study on the Greek population, found lower adherence to pandemic restrictions among patients who were older and had lower levels of education. In the present study, the lower number of referred patients might have been caused by the increased number of telephone counselling sessions instead of traditional examination and the cancelled of routine outpatient visits.

The availability of medical supplies was similar before and during the pandemic. The main reason prompting surgeons to MIGS was required less postoperative follow-up visits than classical surgeries. The pandemic has contributed to the introduction of more such procedures into surgical practice.

This study had limitations. First, it was a single-centre study, which may have been affected by the surgeons’ preferences for particular techniques, which may also vary among centres. Second, we did not analyze cases in which phacoemulsification was performed as a glaucoma treatment (patients with closed-angle glaucoma). It remains unclear how a substantial reduction in the number of performed visual field examinations impacts glaucoma management, as it is the gold standard for the diagnosis and treatment of patients with glaucoma and cannot be replaced by an OCT examination [29]. Moreover, our findings may not be sufficiently generalizable, given that they were obtained from a single centre. More similar studies analyzing the structure of antiglaucoma procedures during the pandemic from other centres are needed. Future studies should also examine whether this factor affects the long-term quality of vision in glaucoma patients.

In conclusion, despite diverting healthcare resources to the treatment of patients with COVID-19 during the COVID-19 pandemic, glaucoma treatment remained relatively accessible. This treatment should be provided safely and responsibly and in compliance with the relevant guidelines, including any infection prevention measures and adaptations to surgical procedures. The COVID-19 pandemic is associated with a decrease in the number of extended procedures and an increase in the number of short procedures performed, such as TSCP and MIGS. This shift may have reduced the number of required postoperative visits and interventions. The analysis of trends in glaucoma surgery during the COVID-19 pandemic may help the ongoing development of treatment guidelines that inform surgical practice and infectious disease prevention procedures. If follow up studies reveal superiority of this new approach, it might be implemented into clinical practice faster than the process would last without the impact of pandemic. The present findings may be useful to clinicians and the ophthalmic society members and directors and for planning the strategy for future waves of the pandemic.

## Data Availability

The data that support the findings of this study are available from the corresponding author [J.K.], upon reasonable request.

## Previous submission

Ophthalmic Epidemiology.
